# Supplementary material for: A single cell characterisation of human embryogenesis identifies pluripotency transitions and putative anterior hypoblast centre
Source: Nat Commun. 2021 Jun 17;12:3679. doi: 10.1038/s41467-021-23758-w (PMC8211662; doi:10.1038/s41467-021-23758-w)
Supplement: Supplementary file 1 — Supplementary Information [file 41467_2021_23758_MOESM1_ESM.pdf]

Supplementary Figure 1

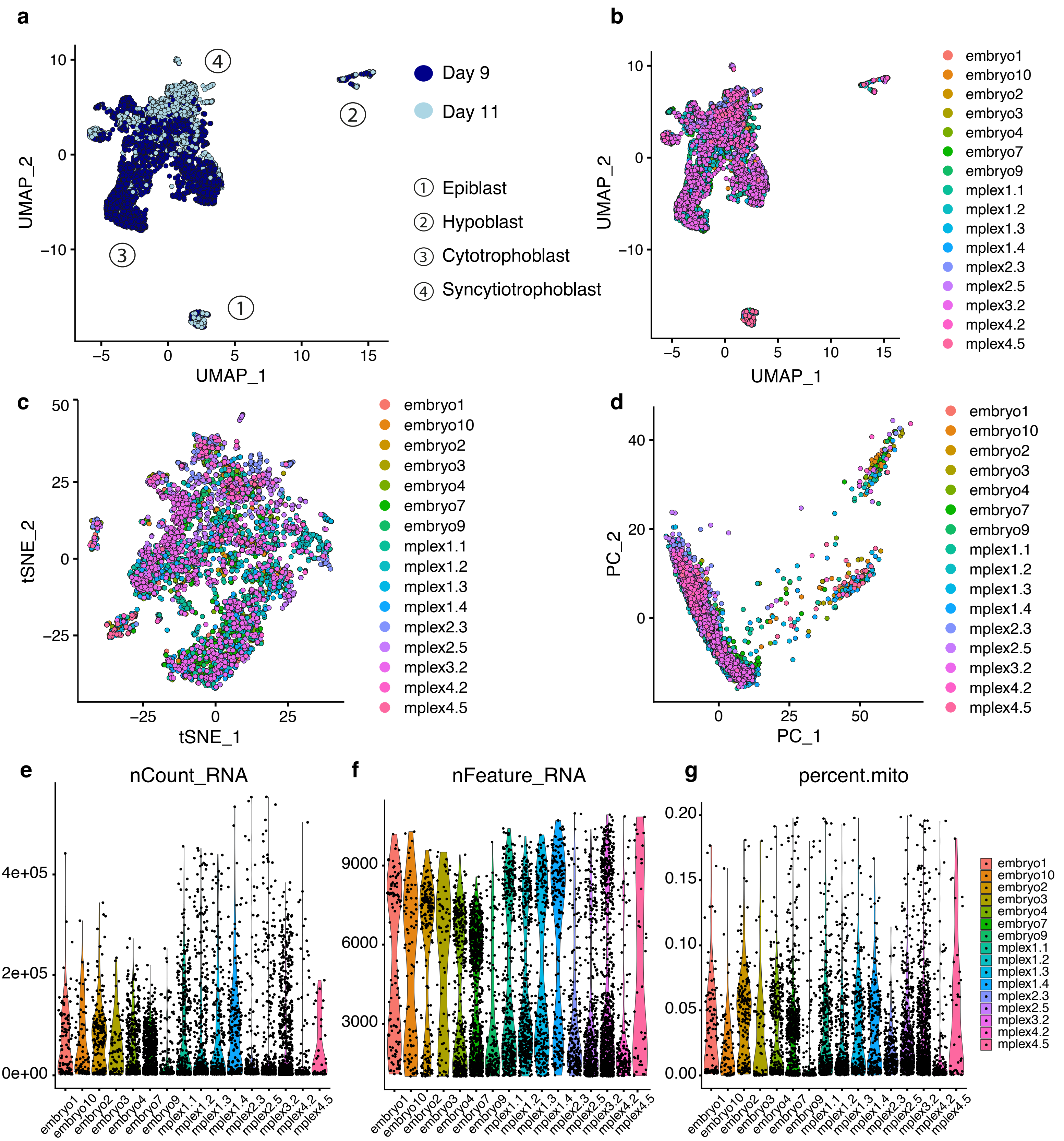

Supplementary Fig. 1: Single cell RNA sequencing data quality control and batch correction. a, UMAP cluster visualisation by embryonic stage: 9 versus 11 d.p.f. Majority of cytotrophoblast cells derive from 9 d.p.f. embryos while syncytiotrophoblast cells from 11 d.p.f. embryos. **b,** UMAP showing distribution of cells colour coded by embryo of origin after batch correction. **c,** tSNE plot of the single cell data coloured by embryo of origin. **d,** PCA plot of the single cell data coloured by embryo of origin. **e,** Violin plots showing the unique molecular identifier (UMI) counts per cell, ordered by embryo number. **f,** Number of features (genes) detected per cell. **g,** Percentage of mitochondrial gene expression across cells. Cells with more than 20% were filtered out.

Supplementary Figure 2

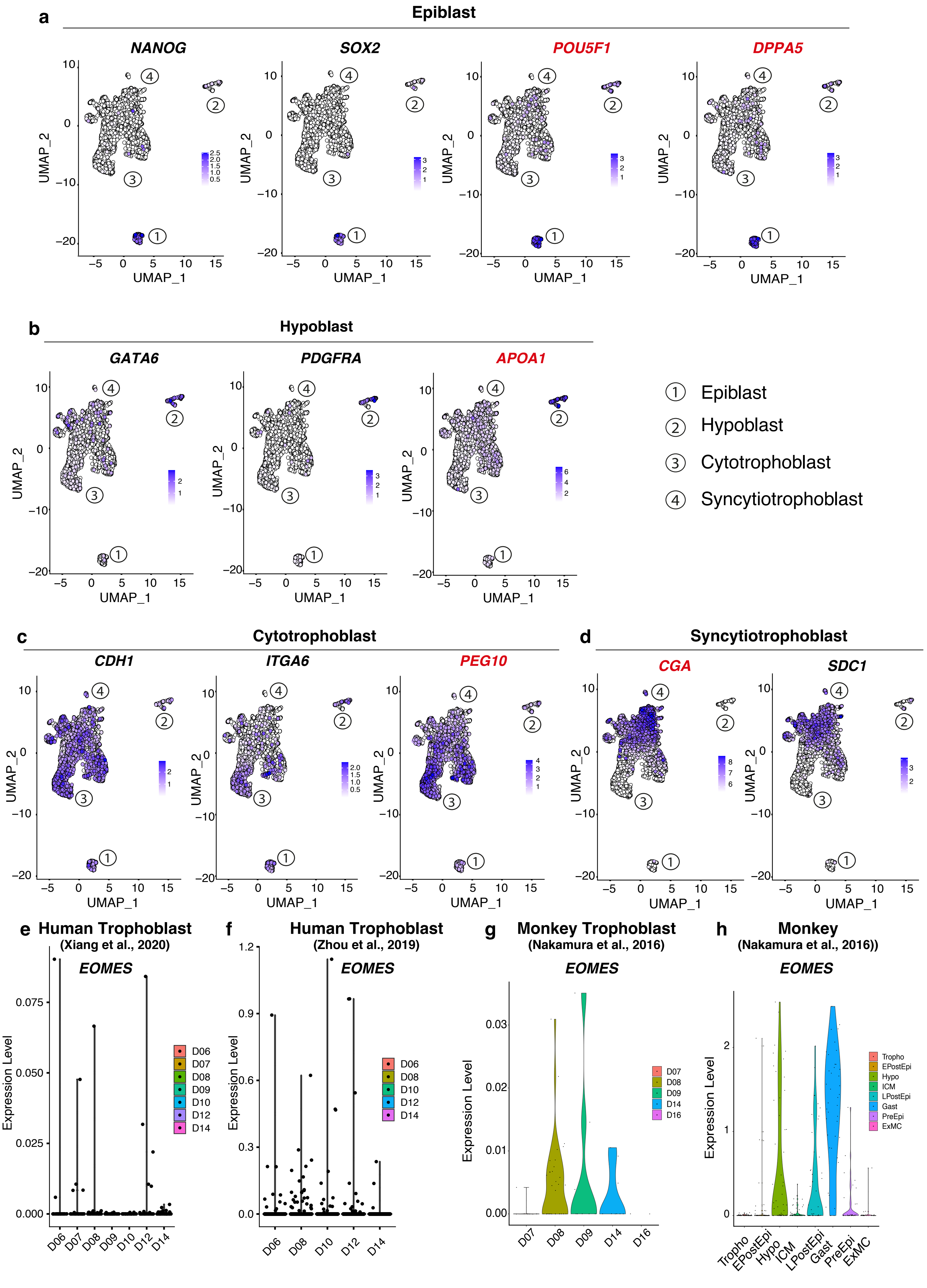

Supplementary Fig. 2: Expression of canonical and enrichened genes. a, The epiblast is characterised by the expression of canonical pluripotency markers *NANOG* and *SOX2*. *POU5F1* and *DPPA5* are the most significantly enriched genes. **b,** The hypoblast is characterised by the expression of canonical markers such as *PDGFRA*, *GATA6. APO1* is the most significantly enriched gene. **c,** The cytotrophoblast is characterised by the expression of markers such as the E-Cadherin gene *CDH1*, the integrin subunit *ITGA6.* Top enriched gene: *PEG10*. **d,** The syncytiotrophoblast is marked by the expression of the markers *CGA* (top enriched) and *SDC1*. **e,** *EOMES* is not expressed in the human trophoblast from D6-D14 in Xiang *et al*., 2020. **f,** *EOMES* is not expressed in the human trophoblast form D6-D14 in Zhou *et al*., 2019[1]. **g-h,** *EOMES* is not expressed in the monkey trophoblast (g) as opposed to other lineages (h) in Nakamura *et al*., 2016.

Supplementary Figure 3

a

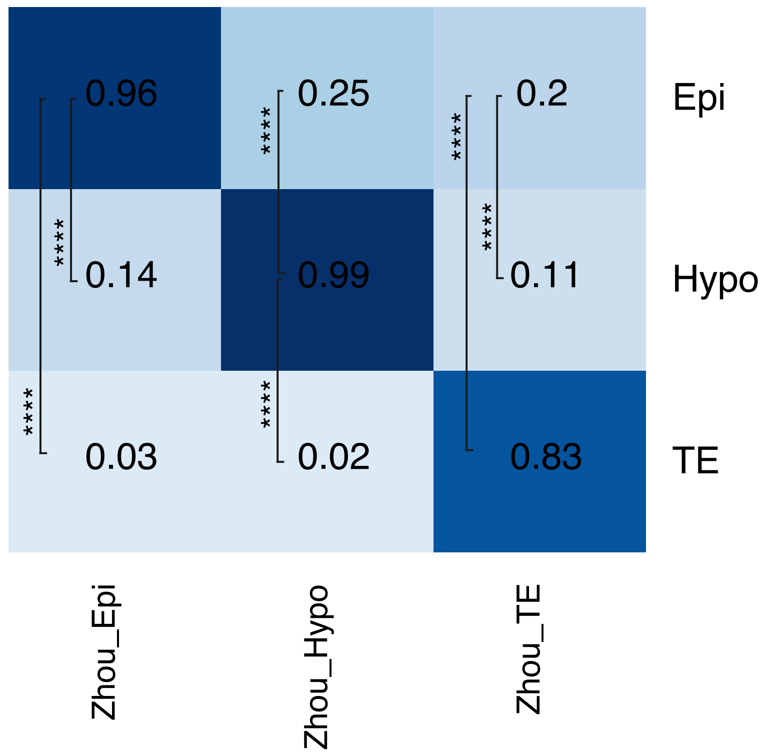

b

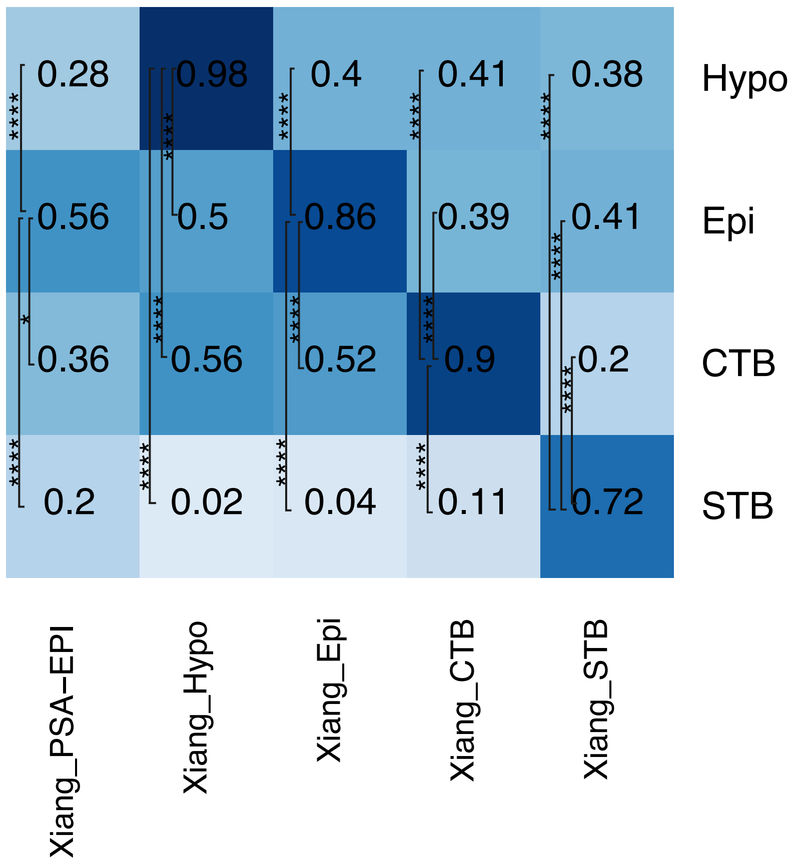

Supplementary Fig. 3: Comparisons with recently published single cell RNAseq datasets. a-b, Logistic regression analysis showing cluster-level similarity scores by heatmap of single cell RNAseq by Zhou et al. 2019[1](**a**) and Xiang et al. 2020[2] (**b**) (x-axis) compared to our dataset (y-axis), which was used as a reference. **a**, Wilcoxon rank sum test (two-tailed) to compare clusters similarities: predicted Epi similarity score for cells in the Zhou_Epi vs Hypo $p < 2.2e^{-16}$, TE $p < 2.2e^{-16}$. Predicted Hypo similarity score for cells in the Zhou_Hypo vs Epi $p < 2.2e^{-16}$, TE $p < 2.2e^{-16}$. Predicted TE similarity score for cells in the Zhou_TE vs Epi $p < 2.2e^{-16}$, Hypo $p < 2.2e^{-16}$. **b**, Wilcoxon rank sum test (2-sided) to compare clusters similarities: predicted Epi score for cells in the Xiang_Epi vs Hypo $p<2.2e^{-16}$, CTB $p<2.2e^{-16}$, STB $< 2.2e^{-16}$. Predicted Hypo score for cells in the Xiang_Hypo vs. Epi $p=1.58e^{-14}$, CTB $p=1.58e^{-14}$, STB $p=1.58e^{-14}$. Predicted CTB score for cells in the Xiang_CTB vs. Epi $p<2.2e^{-16}$, Hypo: $p<2.2e^{-16}$, STB $p<2.2e^{-16}$. Predicted STB score for cells in the Xiang_STB vs. Epi $p<2.2e^{-16}$, Hypo $p<2.2e^{-16}$, CTB $p<2.2e^{-16}$. Predicted Epi score for cells in the Xiang_PSA-Epi vs. Hypo $p=2.98e^{-10}$, CTB $p=0.0262$, STB $= 5.88e^{-10}$.

Supplementary Figure 4

Naïve Pluripotency

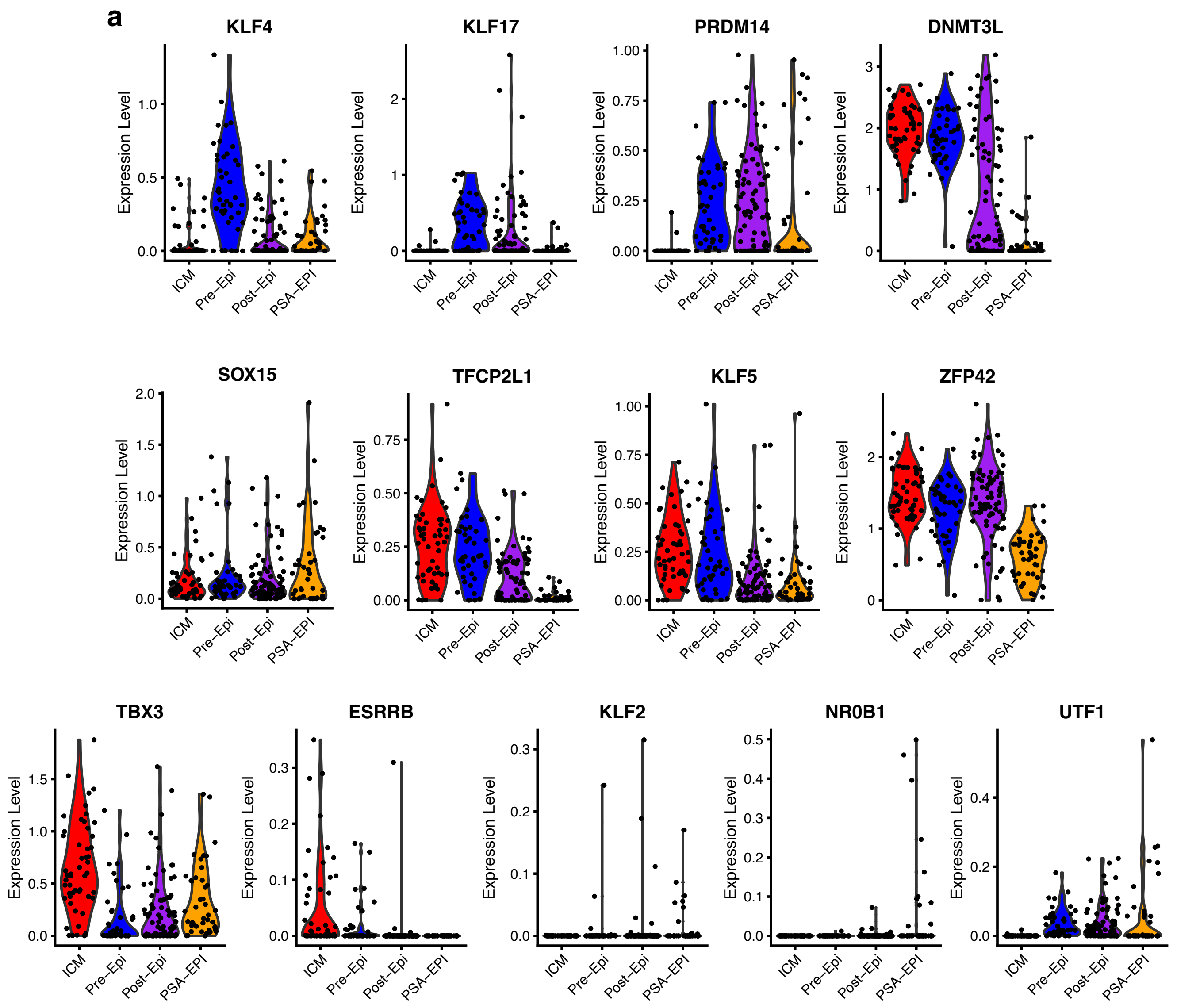

Primed Pluripotency

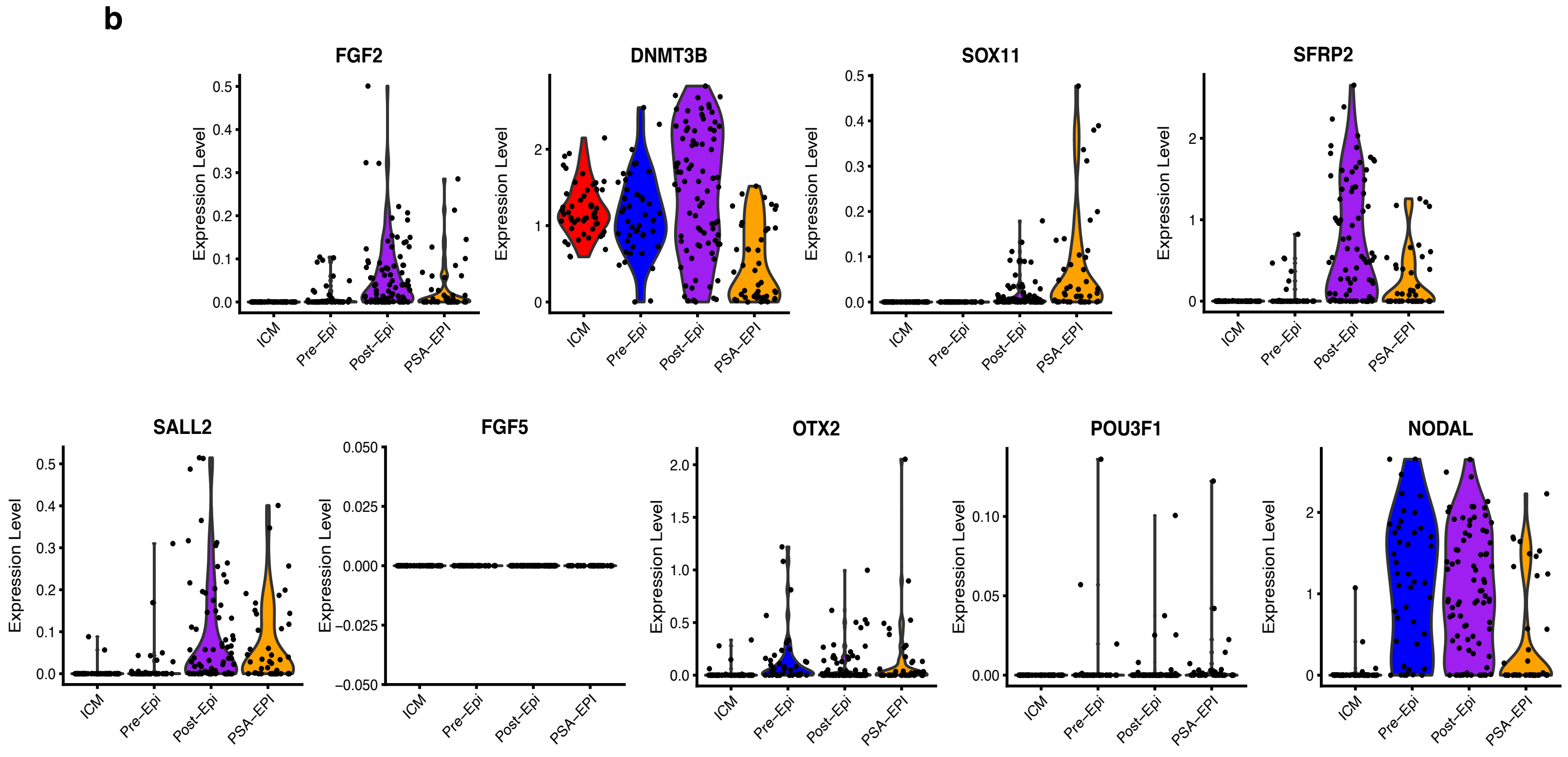

Supplementary Fig. 4: Analysis of the expression of naïve and primed pluripotency markers: Xiang et al. 2020 dataset[2]. a, Naïve genes: *KLF4* and *KLF17* are enriched in pre-implantation epiblast and decrease post-implantation, similarly to our dataset. *ESRRB* is upregulated in the ICM rather than the pre-implantation epiblast. *UTF1* increases in the post-implantation epiblast as shown by our dataset. *DNMT3L*, *TFCP2L1, ZFP42, ESRRB* decrease from pre- to post-implantation, similarly to our dataset. **b,** Primed genes: *SFRP2* and *SALL2* are enriched in the post-implantation epiblast compared to pre-implantation. *FGF5, OTX2* and *POU3F1* are generally low, similarly to our dataset. *FGF2, SOX11* and *DNMT3B* show increase in post-implantation epiblast at 9 d.p.f. *NODAL* is upregulated from ICM to pre-implantation epiblast which stays high in the post-implantation epiblast (9-11 d.p.f.), similarly to our dataset.

Supplementary Figure 5

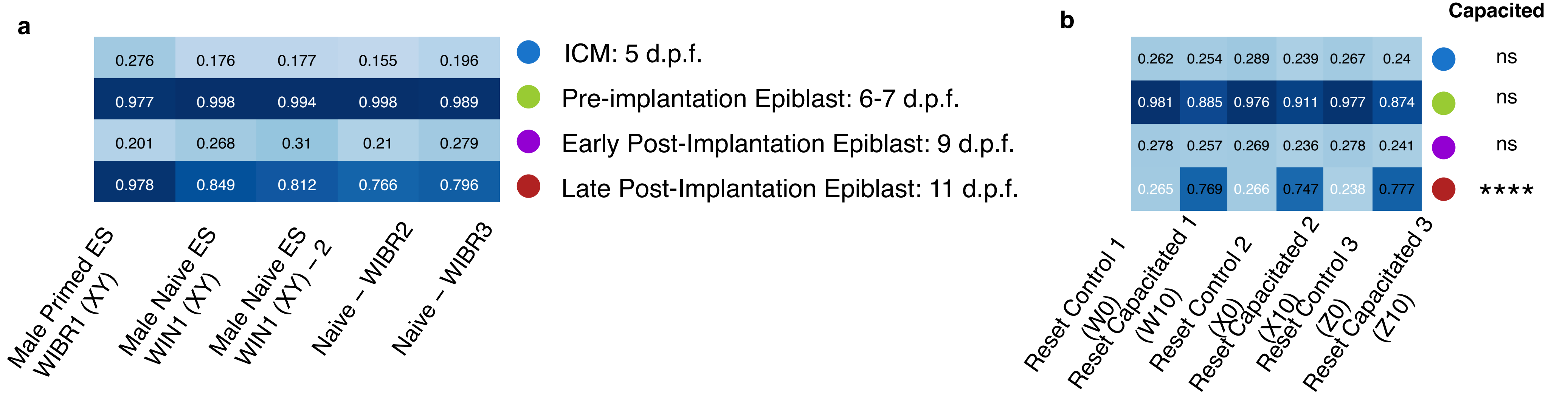

Supplementary Fig. 5: Comparisons between human ESC and human embryos. a, Logistic regression analysis showing quantitative cell matching between human embryo datasets (ICM at 5 d.p.f. (blue), pre-Epi at 6-7 d.p.f.(green) and post-Epi at 9 d.p.f. (purple) and at 11 d.p.f. (red); y-axis) and naïve human embryonic stem cells generated by R. Jaenisch group (Theunissen et al., 2014) [3] (x-axis) . Both primed and naive samples are equally similar to pre-implantation epiblast at 6-7 d.p.f. Naïve cells show high degree of similarity also with the post-Epi at 11 d.p.f., though to a lesser extent than the primed cells **b,** Logistic regression analysis showing quantitative cell matching between human embryo and human ESCs Reset (Reset control) and capacitated (Reset capacitated) generated by A. Smith group (Rostovskaya et al., 2019)[4] (x-axis). Both cells resemble strongly the pre-implantation epiblast at 6-7 d.p.f., while after capacitation Reset capacitated shares similarities with the post-Epi at 11 d.p.f. two-tailed two-way ANOVA w/ Sidak's multiple comparisons: Reset control vs Reset capacitated, 5 d.p.f (p=ns), 6-7 d.p.f ($p<0.0001$), 9 d.p.f ($p=0.0386$) and 11 d.p.f ($p<0.0001$)

Supplementary Figure 6

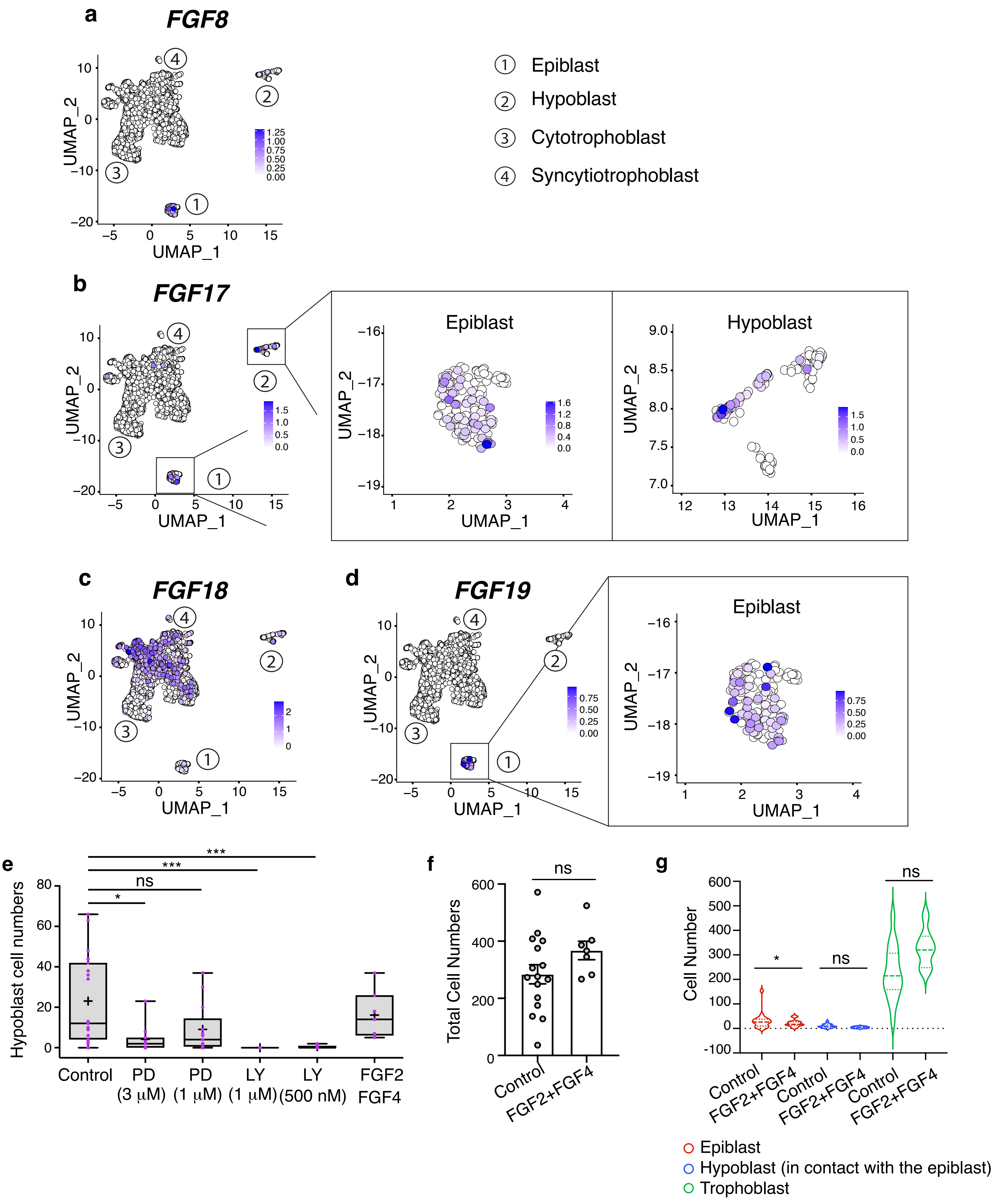

Supplementary Fig. 6: Expression of additional FGF ligands. a-d, In addition to *FGF2* and *FGF4* (Figure 2a), the epiblast is a source of additional FGF ligands: *FGF8* (**a**), *FGF17* (**b**), and *FGF19* (**d**). In contrast, *FGF18* (**c**) is expressed by the sycytiotrophoblast. **e,** Total number of Sox17 positive cells in embryos from Fig. 2c. Two-tailed non-parametric Kruskal-Wallis test with Dunn's correction, *p<0.05, ***p<0.001, ns: non-significant. Source data are provided as a Source Data file**. f,** Total cell numbers in embryos from Fig. 2c. Control group (n=15) and FGF2 + FGF4 treated group (n=7). Two-tailed mann Whitney test, ns: non-significant. Source data are provided as a Source Data file**. g,** Violin plots showing the number of epiblast, hypoblast and trophoblast cells in control and FGF2 + FGF4 treated embryos. Control epiblast (n=18), FGF2 + FGF4 epiblast (n=7), control hypoblast (n=18), FGF2 + FGF4 hypoblast (n=7), control trophoblast (n=16) and FGF2 + FGF4 trophoblast (n=7). Two-tailed F-test for variance, *p=0.045 (test for means by two-tailed Welch's T-test is ns p=031). ns: non-significant. Source data are provided as a Source Data file.

Supplementary Figure 7

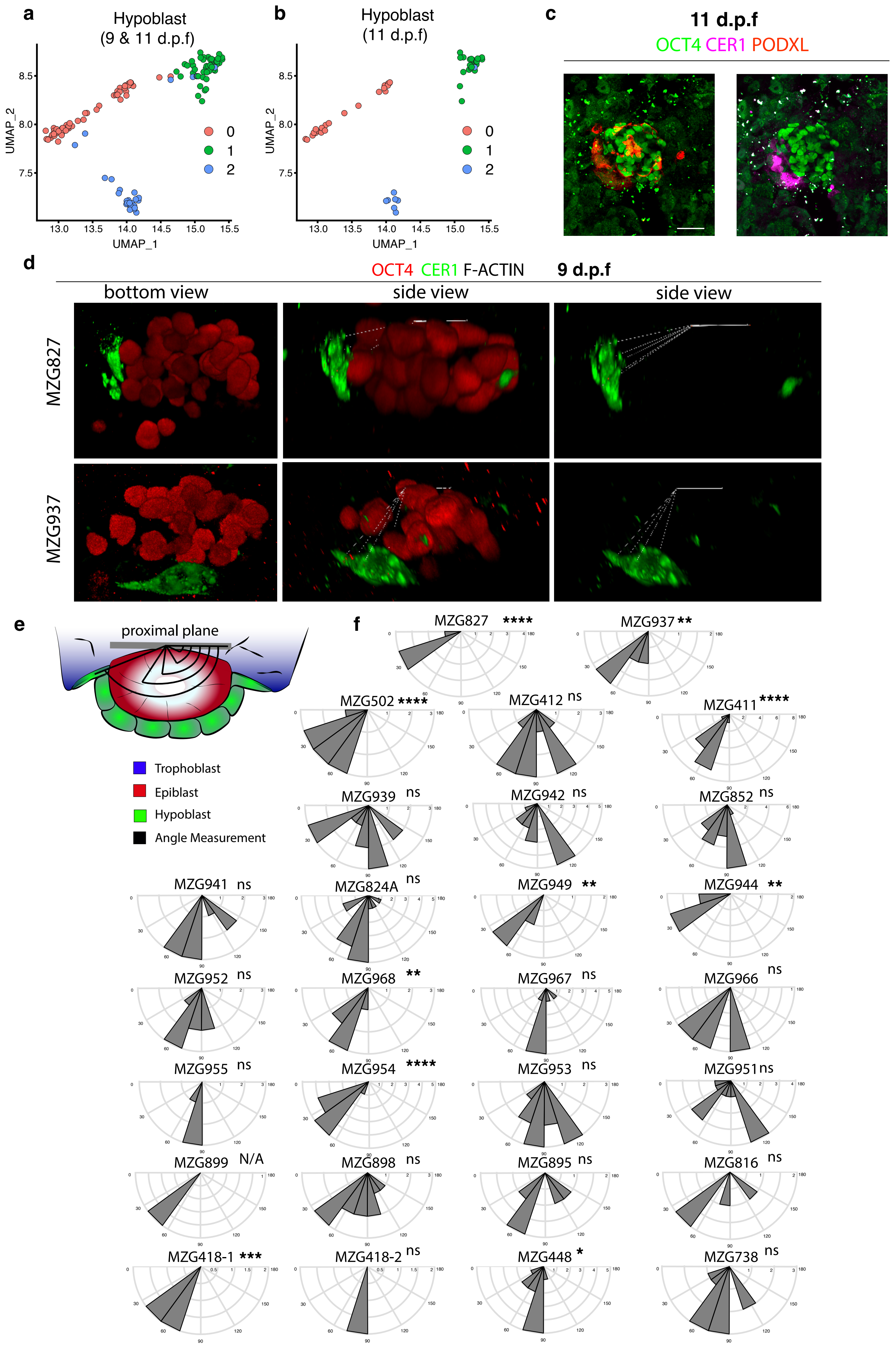

Supplementary Fig. 7: Characterisation of the putative anterior hypoblast signalling centre. a-b, UMAP plot showing a zoom-in view of the hypoblast lineage at 9 and 11 d.p.f combined (**a**) and 11 d.p.f. (**b**), subdivided into three sub-clusters (0-2). The most enriched genes expressed in each sub cluster are fully reported in Supplementary Data 8. **c,** Immunofluorescence analysis of 11 d.p.f. human embryo showing CER1 asymmetric expression. Number of experimental replicates: 2. **d,** Representative 3D reconstruction and orientation of embryos in 3D for the quantification of CER1 positive cells. **e,** Schematic of the method for the quantification of CER1 localisation by 3D viewer. Angles are measured by defining the proximal plane of the epiblast and calculated as frequency distributions from 0° to 180°. **f,** Angular distribution of CER1 positive cells along the hypoblast hemisphere (0° to 180°) calculated for individual embryos at 9 d.p.f. and represented as summary combined data in Figure 3h. Biases in the angular distribution of CER1 cells against the distal plane (90°) for each embryo calculated by two-tailed one sample T-test: 10 embryos are statistically different from 90° degree: MZG949: $p=0.0075$**, MZG944: $p=0.0059$**, MZG937: $p=0.0068$**, MZG827: $p<0.0001$****, MZG968: $p=0.0096$**, MZG954: $p<0.0001$****, MZG418-1: $p=0.005$***, MZG448: $p=0.0112$*, MZG411: $p<0.0001$****, MZG502: $p<0.0001$****; 17 embryos are ns different from 90° degree, p=ns in MZG942, MZG941, MZG939, MZG412, MZG738, MZG816, MZG824-a, MZG418-2, MZG953, MZG967 MZG966, MZG955, MZG 591, MZG952, MZG895, MZG898, MZG852; MZG899: the n number is too small and could not be analysed statistically. Source data are provided as a Source Data file. Scale bar: 50 μm (Fig 7c)

Supplementary Figure 8

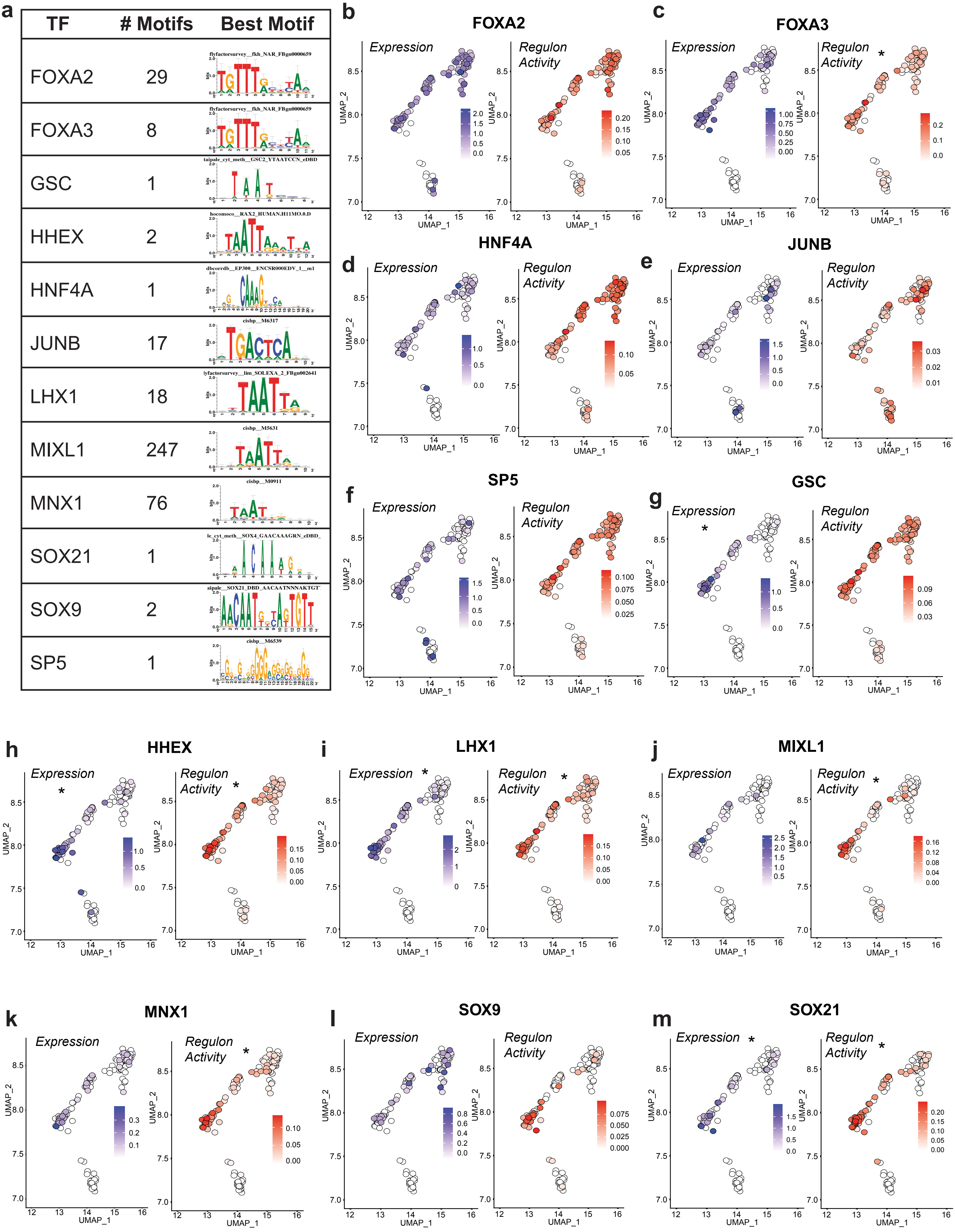

Supplementary Fig. 8: Transcription factors predicted to regulate *CER1* in the putative human anterior hypoblast. a, Table of the transcription factors (TFs) predicted by SCENIC pipeline to regulate *CER1* expression with the number of binding motifs present within 500bp and 10,000 bp of the *CER1* coding sequence, and the best motif candidate identified for each TF. Total 12 TFs identified. **b-m,** Expression (purple scale; Log2(integrated counts+1)) and regulon activity score corresponding to predicted level of transcription factor activity (red scale, Area under the curve (AUC)) for each TF in the hypoblast cluster UMAP projection. Please refer to Figure 3a for the identity of sublcusters 0, 1 and 2. **b,** *FOXA2* **c,** *FOXA3* **d,** *HNF4A* **e,** *JUNB* **f,** *SP5* **g,** *GSC* **h,** <u>*HHEX*</u> **i,** *LHX1* **j,** *MIXL1* **k,** *MNX1* **l,** *SOX9* **m,** *SOX21.* * denotes significantly enriched in subcluster 0 according to ROC analysis.

Supplementary Figure 9

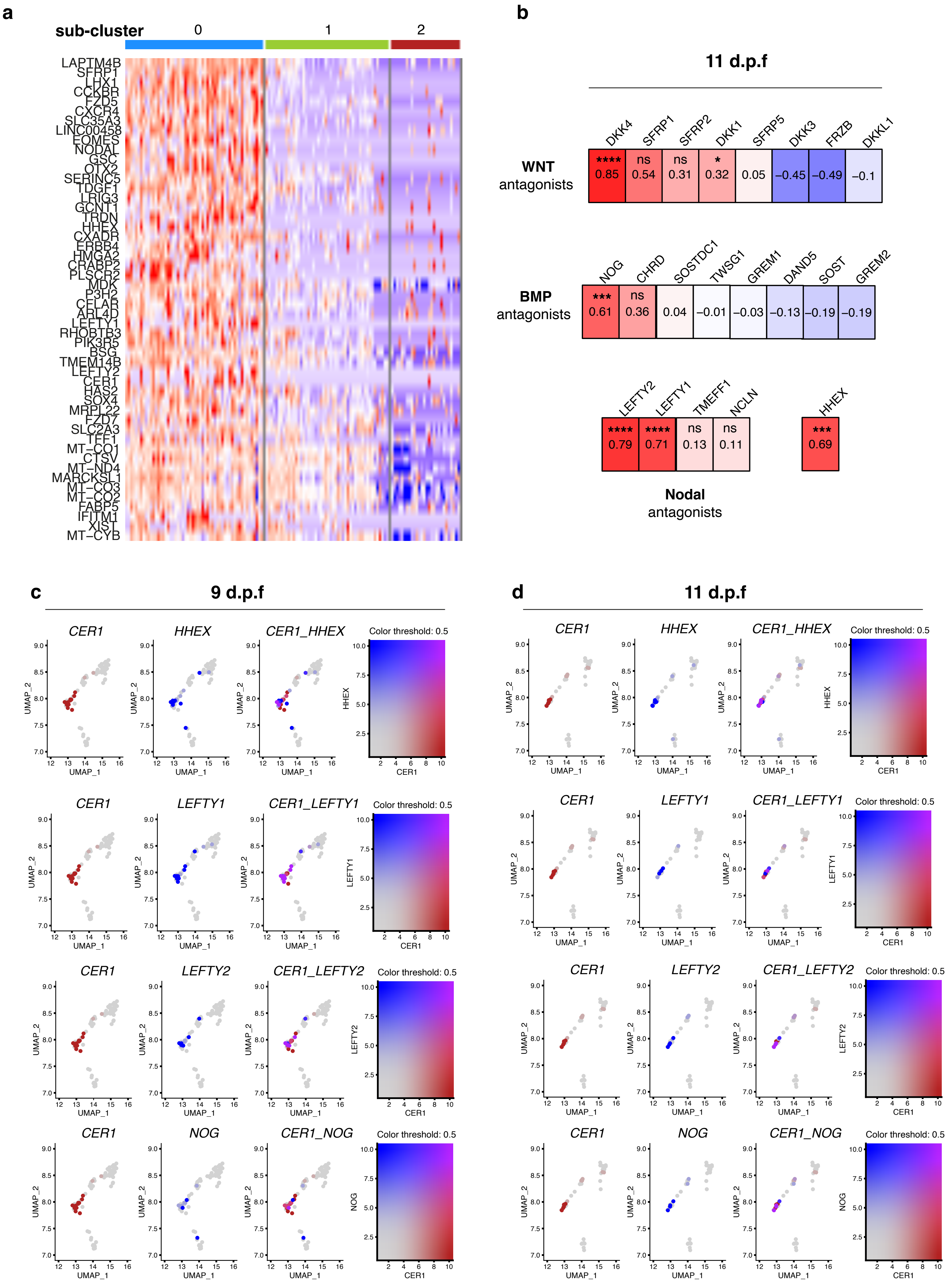

Supplementary Fig. 9: Gene expression profile of the putative anterior hypoblast subcluster. a, Heatmap of the top 50 enriched genes by ROC analysis in hypoblast subcluster 0 as opposed to subclusters 1 and 2. Please refer to Figure 3a for the identity of sublcusters 0, 1 and 2. **b,** Correlation by regression analysis of the expression of CER1 and major WNT, BMP and Nodal antagonists at 11 d.p.f. Co-expression was assessed and Pearson correlation coefficients were tested (two-tailed) and corrected for multiple hypothesis testing with the Benjamini-Hochberg: very significant correlations between *CER1* and *LEFTY1*(p=2.84E^{-17}), *LEFTY2* (p=1.62E^{-16}), *HHEX* (p=0.00011155), *NOG* (p=0.00020516), *DKK4*(p=6.22E^{-07}) and only to minor extent to *DKK1* (p=0.01504934). Correlation *SFRP1*, *SFRP2, CHRD*, *SOSTDC1*, *NCLN*, *TMEFF1* are ns. **c-d,** Feature plots at 9 (**c**) and 11 d.p.f. (**d**) showing expression levels of CER1 (left, red), expression of major WNT, BMP and Nodal antagonists (middle, blue), co-expression between the two genes (right). High level of co-expression between the two genes is displayed in pink, low levels in grey.

Supplementary Figure 10

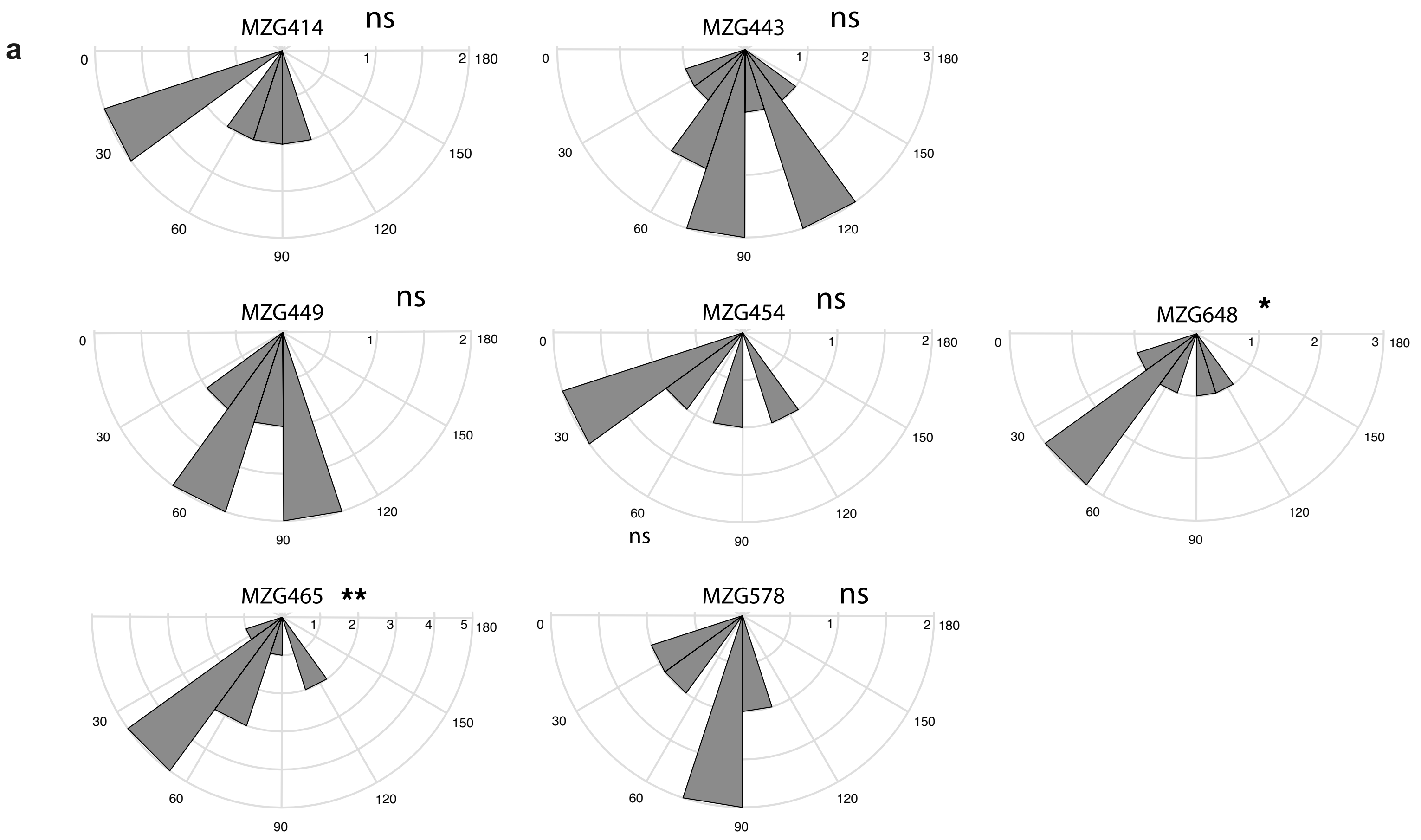

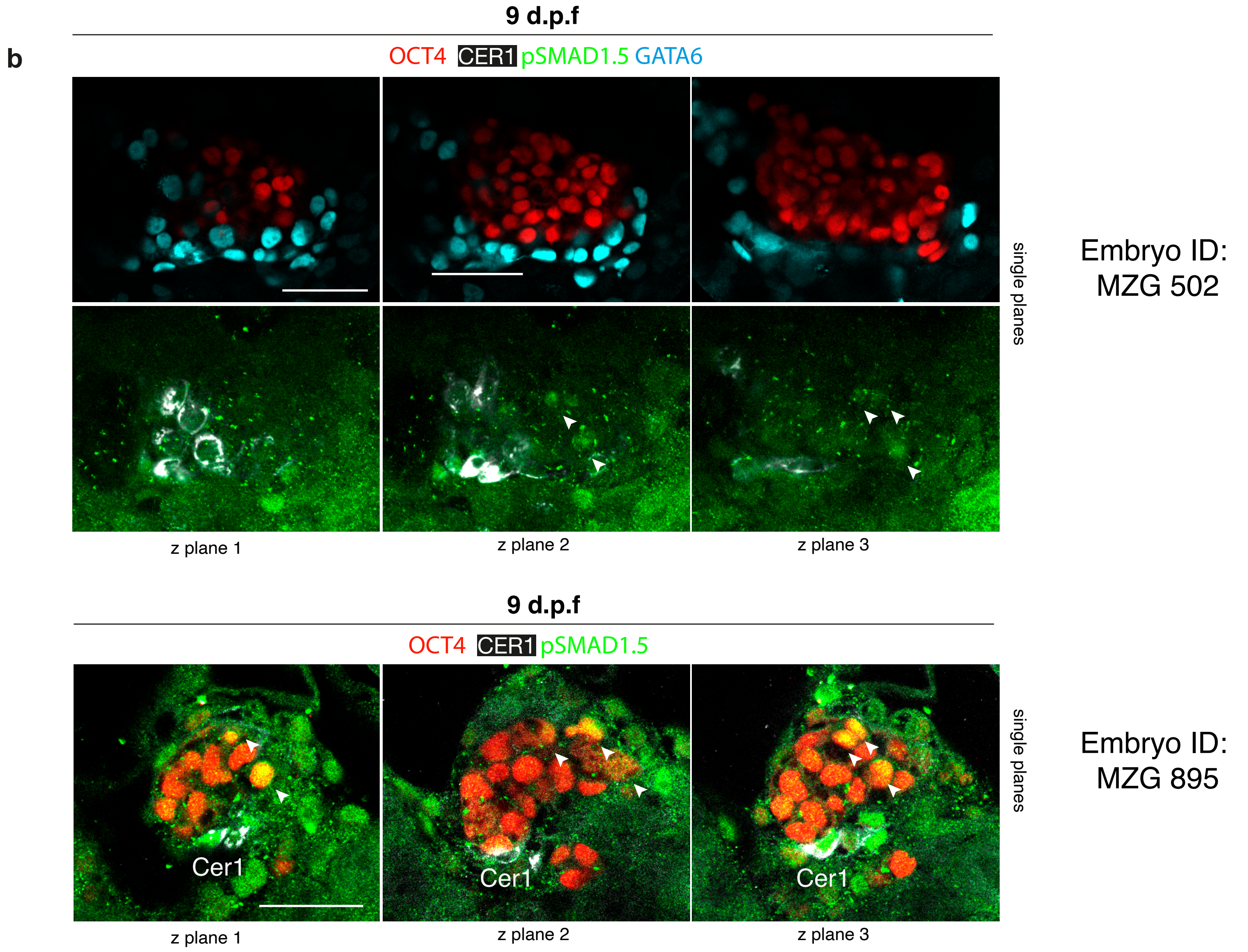

Supplementary Fig. 10: Characterisation of the putative anterior hypoblast signalling (Lefty1) and epiblast patterning. a, Angular distribution of LEFTY1 positive cells along the hypoblast hemisphere (0° to 180°) calculated for individual embryos at 9 d.p.f. and represented as summary combined data in Figure 3l (no of embryos=7). Biases in the angular distribution of LEFTY1 cells against the distal plane (90°) for each embryo calculated by two-tailed one sample T-test: 2 embryos are statistically different from 90° degree: MZG648: p=0.05 *, MZG465: p=0.0094 **; 5 embryos are ns different from 90° degree, p=ns in MZG578, MZG454, MZG449, MZG443 and MZG414. Source data are provided as a Source Data file**. b,** Immunofluorescence analysis at 9 d.p.f. for pSMAD1.5 (BMP pathway) in single z planes. Top embryo is MZG 502 and refers to maximu projection shonw in Figure 3m. Bottom embryo is MZG 895. Subset of OCT4 positive cells expressing nuclear pSMAD1.5 are indicated by arrows. Number of experimental replicates: 8. Total number of embryos stained for pSMAD1.5=29; 9/29 embryos (31%) show nuclear pSMAD1.5 in OCT4 positive cells; 8 out of these 9 embryos (89%) display evident localisation of pSMAD1.5 cells opposite to CER1 localisation. Scale bars: 50 μm (b).

Supplementary Figure 11

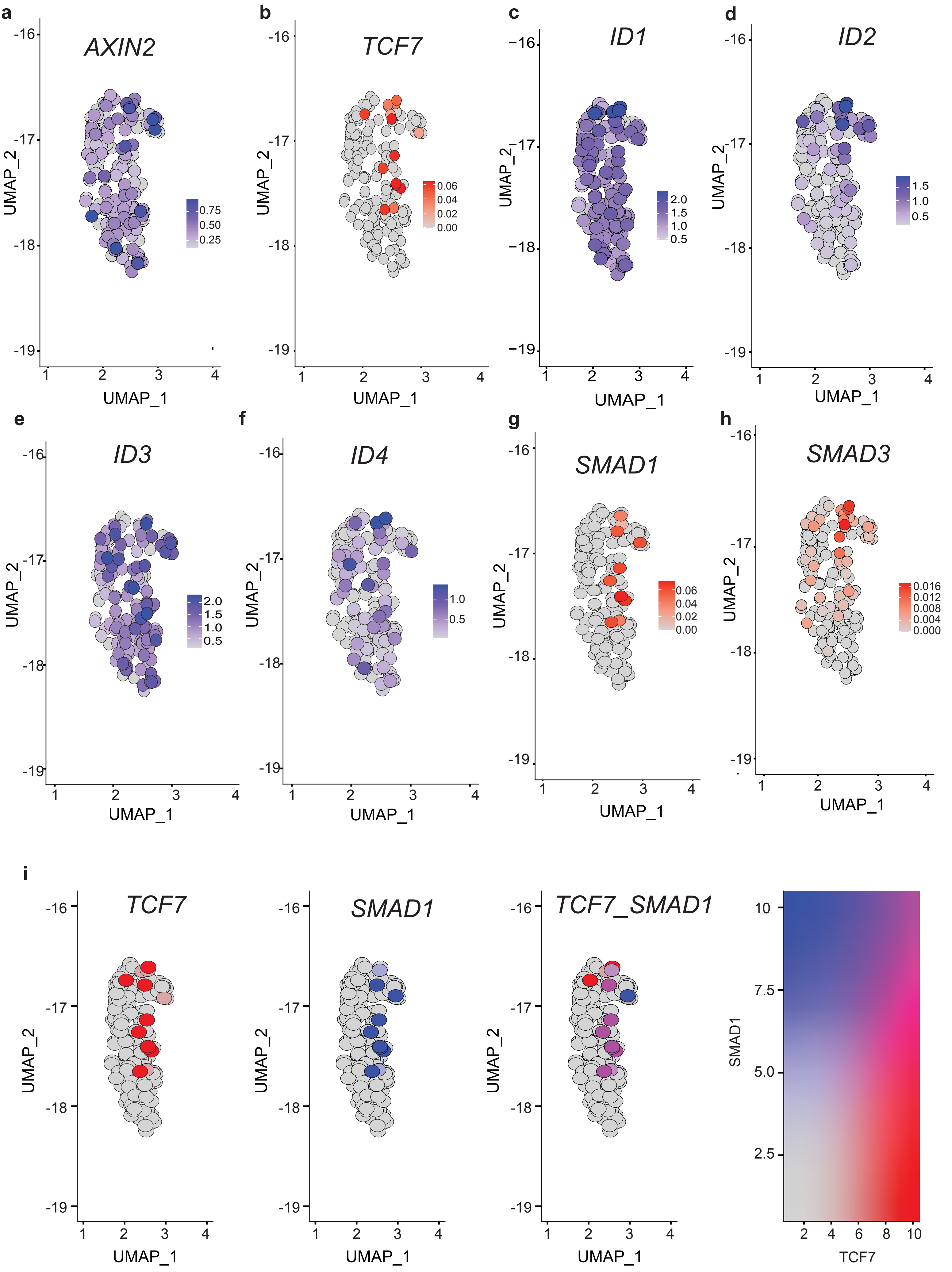

Supplementary Fig. 11: Characterisation of pathways downstream of the putative anterior hypoblast signals. a, Expression in epiblast cluster of WNT target *AXIN2.* **b,** Scored activity by SCENIC in epiblast cluster for WNT transcription factor TCF7 (TCF1). **c-f,** Expression in epiblast cluster of major BMP targets (*ID1-4*). **g,** Scored activity by SCENIC in epiblast cluster of BMP transcription factor SMAD1, **h,** Scored activity by SCENIC in epiblast cluster of Nodal transcription factor SMAD3, **i,** Colocalisation plot of scored TCF7 and SMAD1 activity shows strong overlap in single cells.

Supplementary Figure 12

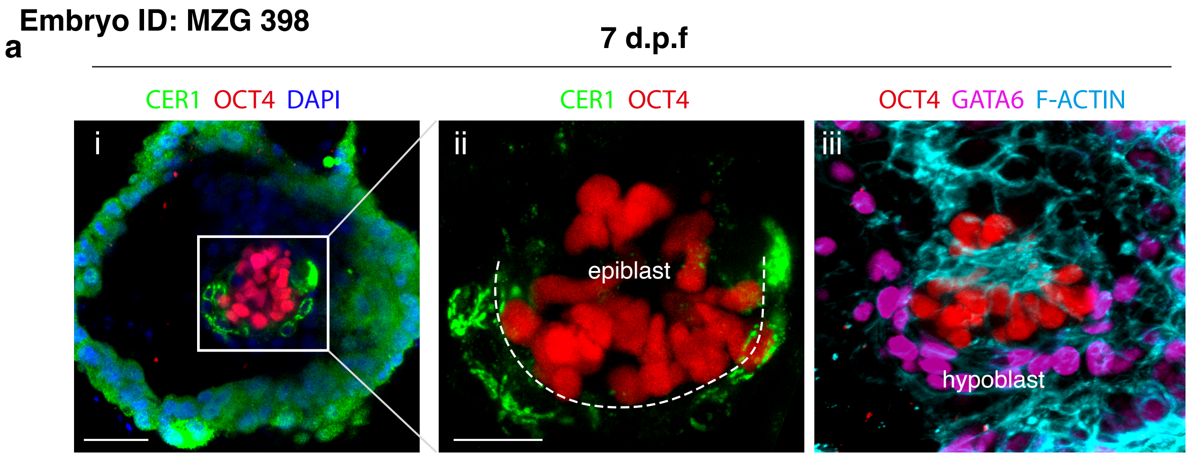

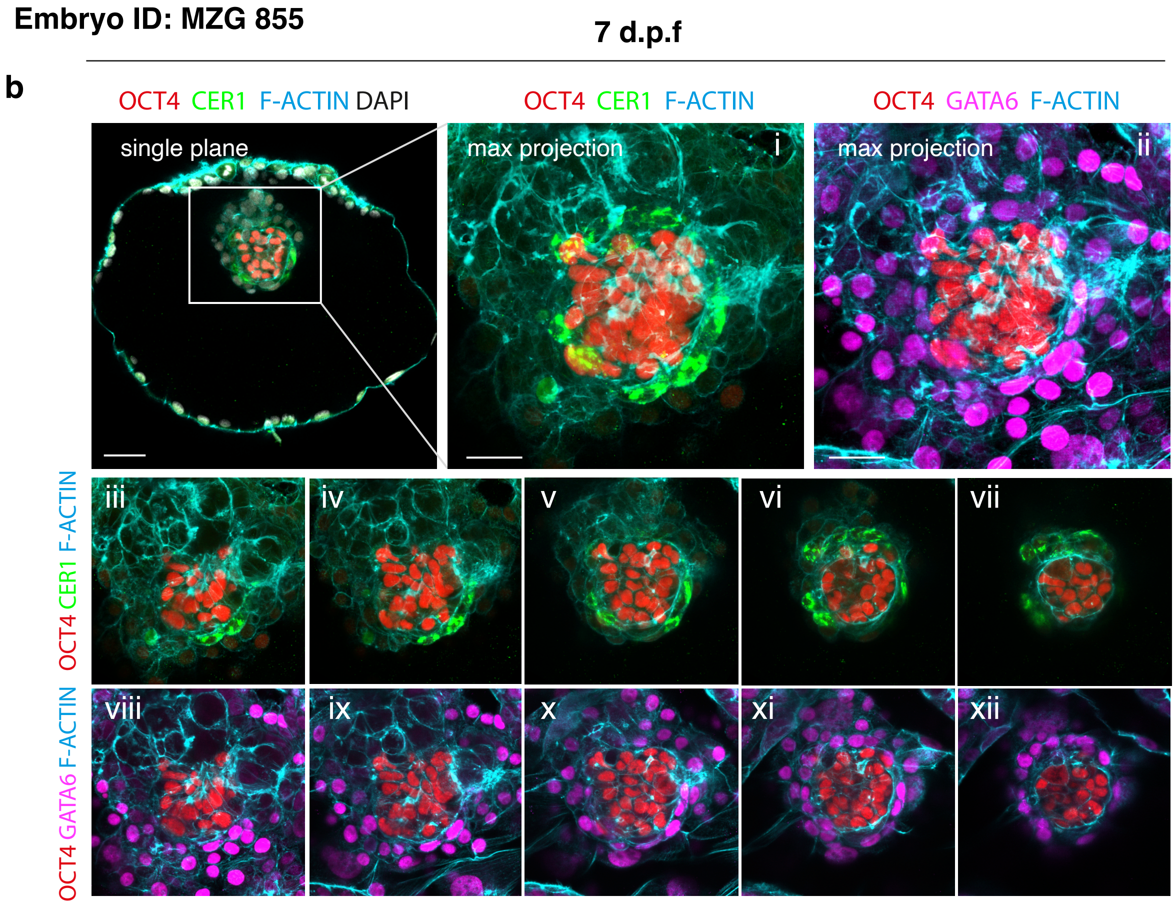

Supplementary Fig. 12: Hypoblast signalling at 7 d.p.f. a-b, Immunofluorescence analysis of embryos at 7 d.p.f. and widespread distribution of CER1 cells along the hypoblast, marked by GATA6+ along the basal distal side of the epiblast epithelium. Number of experimental replicates: 5. Number embryos analysed: 13**.** Scale bars: 50 μm.

Supplementary Figure 13

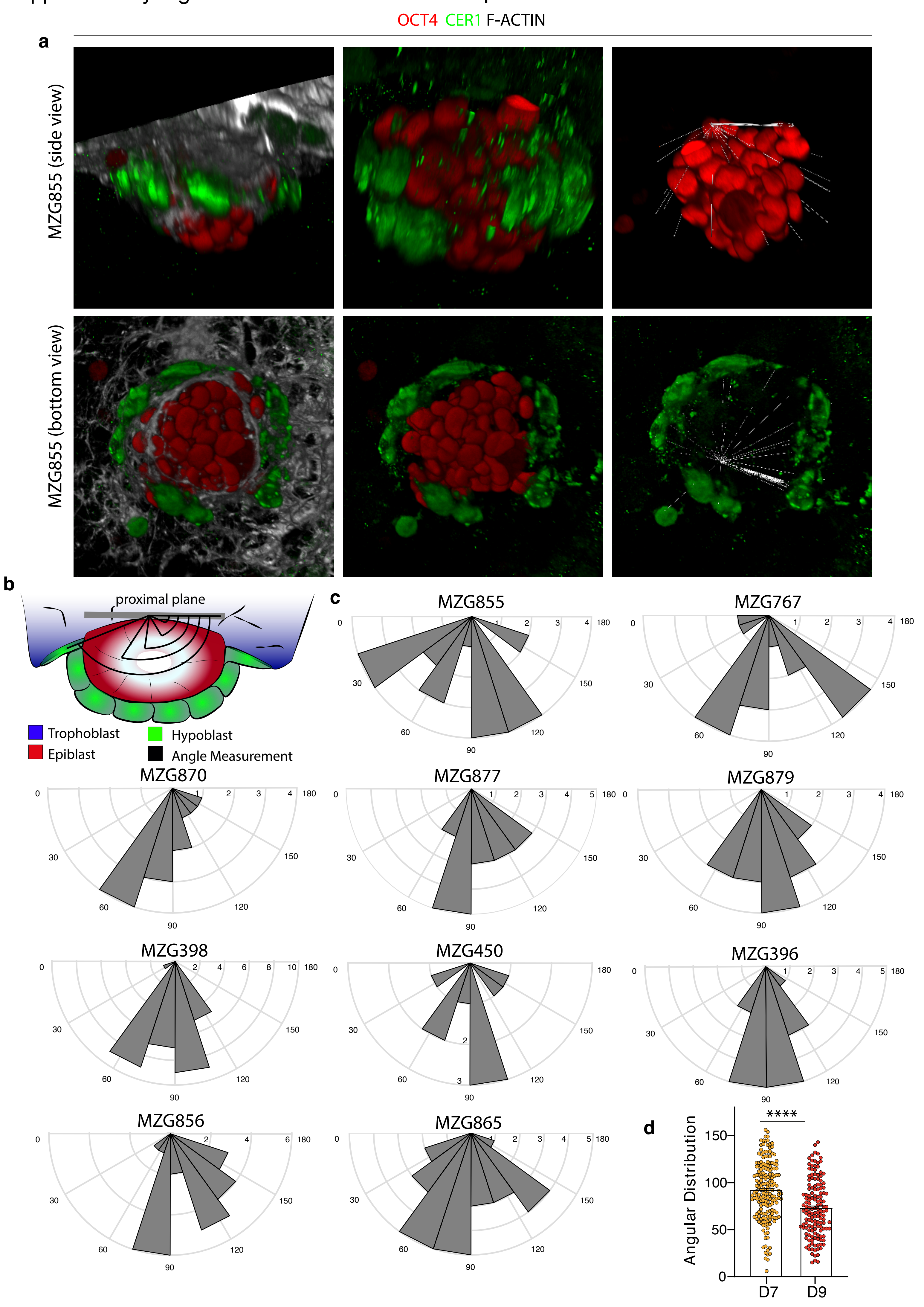

Supplementary Fig. 13: Angular distribution of CER1 positive cells at 7 d.p.f. a, Representative 3D reconstruction and orientation of embryos in 3D for the quantification of CER1 positive cells. **b,** Schematic of the method for the quantification of CER1 localisation by 3D viewer. Angles are measured by defining the proximal plane of the epiblast and calculated as frequency distributions from 0° to 180°. **c,** Angular distribution calculated for each embryo at 7 d.p.f. Difference in the angular distribution of CER1 cells among embryos was analysed by multiple comparisons by the non-parametric Kruskal-Wallis Test: no embryos are significantly different. Source data are provided as a Source Data file. **d,** Angular distribution of CER1 expression in embryos at 7 d.p.f. compared to 9 d.p.f.. box plot shows the mean value whereas single datapoints are representing single cells. The CER1 expressing domain shifts significantly towards one side of the epiblast (92° at 7 d.p.f. to 73° at 9 d.p.f.): two-tailed Welsh's t-test: p=8.83E-06. Source data are provided as a Source Data file.

Supplementary References

1. Zhou, F. *et al.* Reconstituting the transcriptome and DNA methylome landscapes of human implantation. *Nature* **572**, 660–664 (2019).
2. Xiang, L. *et al.* A developmental landscape of 3D-cultured human pre-gastrulation embryos. *Nature* **577**, 537–542 (2020).
3. Theunissen, T. W. *et al.* Systematic Identification of Culture Conditions for Induction and Maintenance of Naive Human Pluripotency. *Cell Stem Cell* **15**, 471–487 (2014).
4. Rostovskaya, M., Stirparo, G. G. & Smith, A. Capacitation of human naïve pluripotent stem cells for multi-lineage differentiation. *Development* **146**, dev172916 (2019).
