# Supplementary material for: A single cell characterisation of human embryogenesis identifies pluripotency transitions and putative anterior hypoblast centre
Source: Nat Commun. 2021 Jun 17;12:3679. doi: 10.1038/s41467-021-23758-w (PMC8211662; doi:10.1038/s41467-021-23758-w)
Supplement: Supplementary file 14 — Description of Additional Supplementary Files [file 41467_2021_23758_MOESM14_ESM.pdf]

Title: Supplementary Data 1.
Description: Human embryos sequenced details. Summary of embryos collected and processed for sc-RNA-seq analysis: 8 embryos at 9 d.p.f. (total of 108 epiblast cells, 82 hypoblast, 1956 CT (cytotrophoblast), 1058 SCT (syncytiotrophoblast)) and 8 embryos at 11 d.p.f (58 epiblast cells, 54 hypoblast ,226 CT, 1278 SCT) (Sheet 1). For each embryo we report the total number of cells analysed per lineage, median counts per cell, median genes per cell. Embryos lacking any of the four lineages were excluded from analysis as indicated by grey highlight in sheet 2 as these represent sub optimally developed embryos. All embryos shown in Sheet 2.

Title: Supplementary Data 2.
Description: Gene enrichment analysis of lineages. Gene enrichment analysis (DEG analysis) by ROC analysis for each lineage: epiblast, hypoblast, cytotrophoblast, syncytiotrophoblast as referred to Figure 1b. Top ten most enriched genes are labelled in red and bold as reported in Fig 1b. myAUC refers to the classifier score for each gene in which 1 means that expression levels of a particular gene can classify the cluster of interest versus all other cells. Avg_diff is the average fold change between the cluster of interest and all other cells. Power is the predictive power of each gene to identify a cells cluster calculated by abs(AUC-0.5)*2.

Title: Supplementary Data 3.
Description: Differential expression analysis 9 vs. 11 d.p.f. Differential expression analysis between scRNAseq dataset at 9 d.p.f. vs. 11 d.p.f. for each lineage by Wilcoxon Ranked Sum test: epiblast, hypoblast, cytotrophoblast, syncytiotrophoblast. p_val is the unadjusted p-value. avg_logFC: log fold-change of the average expression between the two groups (pct1 vs pct2). pct.1: the percentage of cells where the gene is detected in the first group. pct.2: the percentage of cells where the gene is detected in the second group. p_val_adj is adjusted p-value, based on Bonferroni correction. Avg_logFC is the mean log fold change between the two groups.

Title: Supplementary Data 4.
Description: Comparisons published pre-implantation epiblast cells versus post-implantation dataset. Comparisons between pre-implantation single-cell sequenced epiblast cells previously published 5–8 versus our post-implantation dataset. Inner cell mass (ICM): 5 d.p.f. Preepiblast: 6-7 d.p.f. Peri-implantation epiblast: 9 d.p.f. Post-implantation epiblast: 11 d.p.f. The table shows integrated counts for each single cell and its stage. Refer to Figure 1c-f.

Title: Supplementary Data 5. Expression of FGF ligands and receptors.
Description: Analysis of the expression of the FGF ligands and FGF receptors between lineages: epiblast (EPI), hypoblast (HYPO), cytotrophoblast (CTB), syncytiotrophoblast (STB). Expression levels are presented as heatmap as log2 normalised counts (right column), or scaled values (left column). Values were scaled in which the average expression of each gene across all cells is 0 and the variance is 1. Scaled average represents the average of these scaled expression values in which positive numbers denote relative enrichment (higher than average of all cells) while negative numbers denote depletion.

Title: Supplementary Data 6.
Description: Effect of FGF signalling on lineage cell number. Details of the quantification of cell number for each embryo as reported in the graphs presented Figure 2d-f. Epiblast: number of OCT4 positive cells. Hypoblast: number of SOX17 positive cells in contact with the distal basal side of the

epiblast. Trophectoderm: number of nuclei negative for OCT4 or SOX17. Each column corresponds to a different treatment.

Title: Supplementary Data 7.
Description: Quantification of hypoblast cells expressing CER1 at 9 d.p.f. Cell number quantification of CER1 cells, hypoblast cells (marked by the expression of GATA6, only cells in contact with the distal basal side of the epiblast), and percentage of CER1 cells within the hypoblast. Data presented in graphs in Figures 3e-g. Total number of embryos = 19. Embryos cultured from 5 to 9 d.p.f.

Title: Supplementary Data 8.
Description: Analysis of hypoblast sub-clusters. Gene enrichment analysis (DEG analysis) for each subcluster of the hypoblast (Figure 3a-b, Supplementary Figure 9a) by ROC analysis: subclusters 0,1 and 2. Reported are the most enriched genes per each subcluster. myAUC refers to the classifier score for each gene in which 1 means expression levels of that particular gene can classify the cluster of interest versus all other cells. Avg_diff is the average fold change between the cluster of interest and all other cells. Power is the predictive power of each gene to identify a cells cluster calculated by abs(AUC-0.5)*2.

Title: Supplementary Data 9.
Description: Analysis of transcription regulon activity. Analysis of transcription factor (regulon) activity as scored by the SCENIC pipeline for each subcluster of the hypoblast by ROC analysis: subclusters 0,1 and 2. Reported are the most differentially active transcription factors for each subcluser of the hypoblast. myAUC refers to the classifier score for each gene in which 1 means that expression levels of a particular gene can classify the cluster of interest versus all other cells. Avg_diff is the average fold change between the cluster of interest and all other cells. Power is the predictive power of each gene to identify a cells cluster calculated by abs(AUC-0.5)*2. Please refer to methods for further details.

Title: Supplementary Data 10.
Description: Quantification of hypoblast cells expressing CER1 at 7 d.p.f. Cell number quantification of CER1 positive cells, hypoblast cells (marked by the expression of GATA6, only cells in contact with the distal basal side of the epiblast), and percentage of CER1 cells within the hypoblast. Data presented in graphs in Figures 4a-c. Total number of embryos = 13. Embryos cultured from 5 d.p.f. to 7 d.p.f.

Title: Supplementary Movie 1.
Description: 3D reconstruction illustrating zoom-in of embryo stained for DAPI (blue), Phalloidin (white), PODXL (red) and CER1 (green) showing the distribution of CER1 expressing cells. 3D visualisation through the z-stack. The amniotic cavity at the centre of the epiblast and the yolk sac cavity underlying the hypoblast can be detected by PODXL localisation.
