# Supplementary material for: A single cell characterisation of human embryogenesis identifies pluripotency transitions and putative anterior hypoblast centre
Source: Nat Commun. 2021 Jun 17;12:3679. doi: 10.1038/s41467-021-23758-w (PMC8211662; doi:10.1038/s41467-021-23758-w)
Supplement: Supplementary file 15 — Reporting Summary [file 41467_2021_23758_MOESM15_ESM.pdf]

nature research

Corresponding author(s): Magdalena Zernicka-Goetz

Last updated by author(s): May 7, 2021

Reporting Summary

Nature Research wishes to improve the reproducibility of the work that we publish. This form provides structure for consistency and transparency in reporting. For further information on Nature Research policies, see our Editorial Policies and the Editorial Policy Checklist.

Statistics

For all statistical analyses, confirm that the following items are present in the figure legend, table legend, main text, or Methods section.

n/a	Confirmed	
☐	☒	The exact sample size (n) for each experimental group/condition, given as a discrete number and unit of measurement
☐	☒	A statement on whether measurements were taken from distinct samples or whether the same sample was measured repeatedly
☐	☒	The statistical test(s) used AND whether they are one- or two-sided<br>*Only common tests should be described solely by name; describe more complex techniques in the Methods section.*
☐	☒	A description of all covariates tested
☐	☒	A description of any assumptions or corrections, such as tests of normality and adjustment for multiple comparisons
☐	☒	A full description of the statistical parameters including central tendency (e.g. means) or other basic estimates (e.g. regression coefficient) AND variation (e.g. standard deviation) or associated estimates of uncertainty (e.g. confidence intervals)
☐	☒	For null hypothesis testing, the test statistic (e.g. F, t, r) with confidence intervals, effect sizes, degrees of freedom and P value noted<br>*Give P values as exact values whenever suitable.*
☒	☐	For Bayesian analysis, information on the choice of priors and Markov chain Monte Carlo settings
☒	☐	For hierarchical and complex designs, identification of the appropriate level for tests and full reporting of outcomes
☒	☐	Estimates of effect sizes (e.g. Cohen's d, Pearson's r), indicating how they were calculated

Our web collection on statistics for biologists contains articles on many of the points above.

Software and code

Policy information about availability of computer code

Data collection	Single-cell RNA-sequencing was performed using the 10X Genomic Chromium system
Data analysis	Cell Ranger Software suite (version 2.2), Fiji Software (version 2.1.0/1.53c), GraphPadPrism8 (version 9), Kallisto (version 0.44), soupercell (version 2.0), R (version 3.6.1), R package Seurat (version 3.1.5), R package SCENIC (version 1.2.2), matlab (version 9.8.0)

For manuscripts utilizing custom algorithms or software that are central to the research but not yet described in published literature, software must be made available to editors and reviewers. We strongly encourage code deposition in a community repository (e.g. GitHub). See the Nature Research guidelines for submitting code & software for further information.

Data

Policy information about availability of data

All manuscripts must include a data availability statement. This statement should provide the following information, where applicable:

- Accession codes, unique identifiers, or web links for publicly available datasets
- A list of figures that have associated raw data
- A description of any restrictions on data availability

The raw sequencing and expression-count data with cell classifications are deposited at ArrayExpress under accession number E-MTAB-8060. Immunofluorescence data will be available from the corresponding authors upon reasonable request. Datasets can be visualised through the web portals www.humanembryo.org.
The following publicly available datasets were used: Xiang et al., 2020 deposited at Gene Expression Omnibus (GEO) under accession number GSE136447, Zhou et al., 2020 deposited at GEO under accession number GSE109555, Takashima et al., 2014 deposited at ArrayExpress under accession number E-MTAB-2857, Theunissen et al., 2014 deposited at GEO under accession number GSE59435, and Rostovskaya et al., 2019 deposited at GEO under accession number GSE123055.

Field-specific reporting

Please select the one below that is the best fit for your research. If you are not sure, read the appropriate sections before making your selection.

- [x] Life sciences
- [ ] Behavioural & social sciences
- [ ] Ecological, evolutionary & environmental sciences

For a reference copy of the document with all sections, see nature.com/documents/nr-reporting-summary-flat.pdf

Life sciences study design

All studies must disclose on these points even when the disclosure is negative.

Sample size	No sample size calculation was performed. Sample sizes were based on similar studies performed on human embryos (e.g., Fogarty et al, Nature, 2017; Shahbazi et al, Nature, 2017; Gerri et al, Nature, 2020)
Data exclusions	Embryos were excluded from final analysis if they did not contain all three lineages or low gene coverage as reported in Extended data Table 1. For immunofluorescence and quantification analyses, embryos that died during culture were excluded from further analysis as well as those that did not develop an epiblast.
Replication	To ensure reproducibility of the results, each experiment was carried out at least 3 times independently. All attempts at replication were successful.
Randomization	The sample allocation was randomised.
Blinding	The majority of the experiments performed are descriptive, and therefore samples were not assigned to experimental groups. For inhibitor treatment experiments, control untreated and experimental treated groups were analysed. Blinding was not possible, as in many cases inhibitor-treatment embryos were easily distinguished based on morphological criteria. For all groups, healthy samples of representative stages and status were allocated to ensure comparability.

Reporting for specific materials, systems and methods

We require information from authors about some types of materials, experimental systems and methods used in many studies. Here, indicate whether each material, system or method listed is relevant to your study. If you are not sure if a list item applies to your research, read the appropriate section before selecting a response.

Materials & experimental systems

n/a	Involved in the study	
☐	☒	Antibodies
☒	☐	Eukaryotic cell lines
☒	☐	Palaeontology and archaeology
☒	☐	Animals and other organisms
☐	☒	Human research participants
☒	☐	Clinical data
☒	☐	Dual use research of concern

Methods

n/a	Involved in the study	
☒	☐	ChIP-seq
☒	☐	Flow cytometry
☒	☐	MRI-based neuroimaging

Antibodies

Antibodies used	The following primary antibodies were used: goat polyclonal anti CERBERUS1 (AF1075, R&D Systems), goat polyclonal anti LEFTY (AF746, R&D Systems), mouse monoclonal anti OCT3/4 (sc-5279, clone C-10, Santa Cruz Biotechnology), mouse monoclonal anti PODOCALYXIN (MAB1658, clone 222328, R&D Systems) and goat polyclonal anti SOX17 (AF1924, R&D Systems). Where indicated secondary antibodies were incubated together with Alexa Fluor 488/584 phalloidin (A12379, ThermoFisher Scientific, dilution 1/500) and DAPI (D3571, ThermoFisher Scientific, dilution 1/1000).
Validation	The antibodies used in this study have been extensively used by the laboratory of Magdalena Zernicka-Goetz. The validation has been done using different methologies and published in different articles as outlined below:<br>- CERBERUS1: Kyprianou et al, Nature, 2020 and Amadei et al, Developmental Cell, 2021: validated by immunofluorescence in mouse embryos and ESCs.<br>- LEFTY: Amadie et al, Developmental Cell, 2021: validated by immunofluorescence in ESCs.<br>- OCT3/4: Shahbazi et al, Nature, 2017: validated by immunofluorescence in Oct4 siRNA-treated cells<br>- PODOCALYXIN: Shahbazi et al, Nature, 2017: validated by immunofluorescence in Podocalyxin KO ESCs<br>- SOX17: Bolton et al, Nature Communications, 2016: validated by immunofluorescence in mouse embryos<br>In addition, these antibodies have been used by several other groups in the mammalian embryo field and are cited in multipe publications.

Human research participants

Policy information about studies involving human research participants

Population characteristics	Surplus embryos donated from patients undergoing IVF treatment were used. The stage of the embryos ranged from Day 5 to Day 6 post fertilisation.
Recruitment	Through IVF clinics: CARE fertility group, Bourn Hall, Herts & Essex. Patients with supernumeray human embryos are offered the possibility of donating them for research. Patients are not pre-selected based on any specific criteria.
Ethics oversight	Approved by the ethical committee of the University of Cambridge and the HFEA.

Note that full information on the approval of the study protocol must also be provided in the manuscript.
